# Factors associated with the use of oral health services in Peruvian children under the age of 12 years

**DOI:** 10.1002/cre2.674

**Published:** 2022-10-27

**Authors:** José Diego Torres‐Mantilla, Edda E. Newball‐Noriega

**Affiliations:** ^1^ Unidad de Salud Pública, Escuela de Posgrado, Universidad Peruana Unión (UPeU) Lima Perú; ^2^ Departamento de Odonto‐estomatología Hospital Carlos Lanfranco la Hoz Lima Perú; ^3^ Departamento de Ciencias Básicas Escuela de Medicina Humana, Facultad de Ciencias de la Salud, Universidad Peruana Unión (UPeU) Lima Perú

**Keywords:** health services accessibility, minors, oral health, Peru, public health dentistry

## Abstract

**Objectives:**

To determine the prevalence and factors associated with the use of oral health services in Peruvian children under 12 years of age.

**Material and Methods:**

A secondary analysis of 2019 Demographic and Family Health Survey was conducted. The sample consisted of 40,751 children. The main variable was the use of dental services (attended/not attended) in the last 6 months, and the independent variables were gender, age, area of residence, wealth quintile, health insurance coverage, information received on oral health care, age, and educational level of the caregivers. Analyses of absolute and relative frequencies, differences in proportions, and multivariate analysis using generalized linear models were performed.

**Results:**

The dental service utilization prevalence during the last 6 months was 31%. Correlation was found with urban area residents (PRa = 0.945; 95% CI: 0.904–0.988), the Jungle geographical domain (PRa = 0.926; 95% CI: 0.877–0.977), the highest wealth quintile (PRa = 1.323; 95% CI: 1.232–1.421), the higher education level of the caregiver (PRa = 1.375; 95% CI: 1.231–1.536), affiliation with the Public Health Insurance (PRa = 1.112; 95% CI: 1.069–1.158), and the condition of having received information on oral health care (PRa = 2.355; 95% CI: 2.263–2.245) with respect to their baseline variables.

**Conclusions:**

Several socio‐demographic factors were correlated with the use of oral health services in Peruvian children under 12 years of age and the percentage of their use was low. Information on oral health care had a more significant impact on both, the population from the highest wealth quintile and the highest educational attainment.

## 1 INTRODUCTION

According to 2017 Global Burden of Disease estimates, approximately 3.5 billion people worldwide suffer from diseases of the oral cavity and caries in permanent teeth is recorded as the most prevalent condition (James et al., 2018). Such prevalence has been consistent, to the extent that caries incidence is universally used as an indicator of oral health status and strategies for improvement involve the prevention and treatment of dental caries (Glick et al., 2021; Hobdell et al., 2003).

In the pediatric population, World Health Organization estimates indicate that dental caries affects 60%–90% of school‐aged children (Petersen et al., 2005), which demonstrates the need to address the high global prevalence of childhood caries as a public health problem. Therefore, in search for epidemiological bases to guide oral health improvement and care programs, several studies have identified the experience of caries during childhood as a risk factor for the development of the disease in the future (Chaffee et al., 2017; Zemaitiene et al., 2016).

The association between the use of oral health services and improved oral health status has been established when comparing similar socio‐demographic backgrounds (Alsuraim & Han, 2020; Botello‐Harbaum et al., 2012; Fisher‐Owens et al., 2016). Likewise, various studies indicate that the Social Determinants of Health (SDOH) are associated in different ways with the use of oral health services, according to the particular characteristics of each population (Aravena‐Rivas & Carbajal‐Rodríguez, 2021; Azañedo et al., 2019; Dho, 2018; Hernández‐Vásquez et al., 2019; Kino et al., 2019). Thus, in the pediatric population, factors such as higher family socioeconomic status increase the likelihood of accessing these services (Gao et al., 2020; Nazir, 2018; Onyejaka et al., 2016; Portero de la Cruz & Cebrino, 2020); while others, such as oral health literacy, offer controversial results (Adil et al., 2020; Baskaradoss et al., 2019; Firmino et al., 2017; Firmino, Ferreira, et al., 2018; Firmino, Martins, et al., 2018; Quadri et al., 2018).

In Peru, the most recent nationwide study indicated a prevalence of 85.6% of dental caries in schoolchildren aged 3–15 years (Ministerio de Salud, 2017). Furthermore, dental caries was the second most common diagnosis reported in outpatient visits nationwide in 2019, with 42.5% of cases occurring in children under 12 years of age (Ministerio de Salud, 2019a), demonstrating the need for improved oral health promotion, prevention, and management of oral health in Peruvian children.

However, there is a lack of sufficiently updated and representative information that analyzes the socio‐demographic data of a child population such as the Peruvian one, regarding its relationship with the use of oral health services, which could contribute to the understanding of the limitations inherent to population and the consequent development of more efficient oral health public policies.

For these reasons, the aim of this study was to evaluate the prevalence and factors associated with access to oral health services among children under 12 years of age in Peru, based on data from 2019 Demographic and Family Health Survey of Peru (ENDES).

## 2 METHODS

An observational, analytical, and cross‐sectional study was conducted from a secondary analysis of data obtained from the ENDES, implemented with a national scope by the National Institute of Statistics and Informatics of Peru (INEI) in 2019. The ENDES collects up‐to‐date information on aspects of the Peruvian population, such as demographic dynamics and factors associated with health status, through household interviews conducted in nationally representative samples. It is a two‐stage, probabilistic, balanced sample type, stratified, and independent survey at the departmental level and by the urban–rural area; it uses households as the sampling unit and persons residing in the household as the unit of analysis. The sample for the year 2019 was 36,760 dwellings.

**Figure 1:**
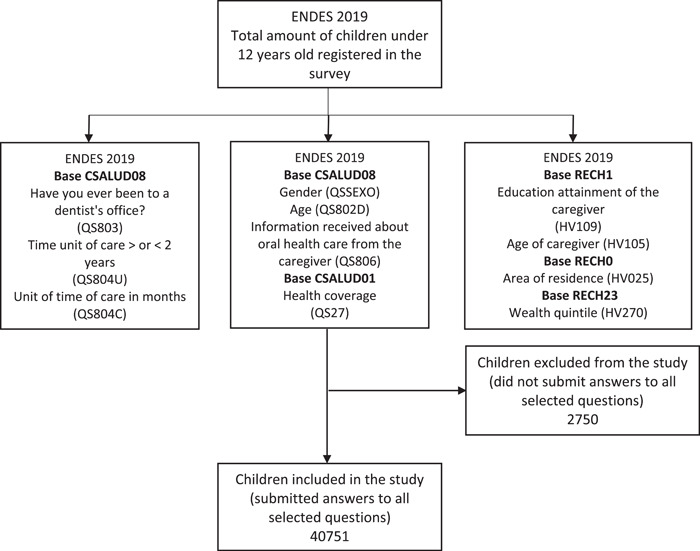
Flow chart showing the screening of children under 12 years old included in the study. Demographic and Family Health Survey (ENDES) 2019.

The ENDES 2019 recorded a total of 43,501 children under 12 years of age; from which a sample of 40,751 was selected, which included only those who submitted complete data on all variables assessed in this study (Figure 1).

The dependent variable analyzed was the use of oral health services in the last 6 months, dichotomized (attended/not attended) from questions QS803, QS804U, and QS804C of the Health Questionnaire in children aged 0–11 years, concerning the date of the last dental care; while the independent variables were age, gender, wealth quintile (classified into five levels of wealth), area of residence (classified as urban and rural), geographic domain (classified as Metropolitan Lima, Rest of coast, Highlands, and Jungle), health coverage (classified as Public Health Insurance [SIS], Direct Contribution Insurance for formal employees: ESSALUD, Direct Contribution Insurance for Armed and Police Forces, Private Insurance, and Uninsured), educational level, and age of the caregiver and information received in the last 12 months on oral care and hygiene (caregiver received/not received). The characterization and coding of the variables can be found in the Health Dictionaries of the Health Survey, Household Characteristics, and Housing Characteristics modules of the ENDES 2019 (http://iinei.inei.gob.pe/microdatos/).

Data analysis was carried out using Stata® v15.1 statistical software (Stata Corporation). For the descriptive analysis of the variables, absolute and relative frequencies were used; while to assess their association with the use of oral health services, an analysis of the difference of proportions was applied through the estimation of *χ* ^2^, with a *p* < .05. Generalized linear Poisson models, the relationship of the dependent variable through the log link function and robust variance estimates in a crude and adjusted model, with 95% confidence intervals and a *p* < .05 considered statistically significant, were also used to determine their correlation. An independent model was also conducted to assess the correlation between variables related to child caregivers and having received oral health care information, given the high correlation of this variable with the use of oral health services.

The present research did not require the approval of an Ethics Committee, as it consists of a secondary analysis of a public domain database, available at PERÚ Instituto Nacional de Estadística e Informática (inei. gob. pe).

## 3 RESULTS

Most of the children included in the study were between 6 and 11 years of age (40%), lived in urban areas (67.1%), were affiliated with the Public Health Insurance SIS (56.5%), and belonged to the lowest wealth quintile (31.6%); while the most populated geographic domain was the Highlands (33.4%). The highest percentage of caregivers reached the Secondary level of education (44.6%) and received information on oral health care (62.2%) (Table 1).

**Table 1:** Characteristics of children under 12 years of age who attended dental services

Variable		Children under 12 years old		Use of oral health services in children under 12 years in the last 6 months		
		*n*	Proportion	Yes	No	*p*
Total		40,751	100%	31% (12628)	69% (28123)	
Age	6–11 years old	16,299	40.0%	5924 (36.3%)	10,375 (63.7%)	.000
	3–5 years	12,081	29.6%	4200 (34.8%)	7881 (65.2%)	
	0–2 years	12,371	30.4%	2504 (20.2%)	9867 (79.8%)	
Gender	Male	20,787	51.0%	6359 (30.6%)	14,428 (69.4%)	.077
	Female	19,964	49.0%	6269 (31.4%)	13,695 (68.6%)	
Caregiver received oral health information	Yes	25,360	62.2%	10,204 (40.2%)	15,156 (59.8%)	.000
	No	15,391	37.8%	2424 (15.7%)	12,967 (84.3%)	
Age of the caregiver	70 years and older	88	0.2%	18 (20.5%)	70 (79.5%)	.000
	50–69 years old	1275	3.1%	344 (27%)	931 (73%)	
	30– years old	24,303	59.6%	7696 (31.7%)	16,607 (68.3%)	
	15–29 years old	15,085	37.0%	4570 (30.3%)	10,515 (69.7%)	
Educational level of the caregiver	Higher education	11,622	28.5%	4393 (37.8%)	7229 (62.2%)	.000
	Secondary	18,175	44.6%	5514 (30.3%)	12,661 (69.7%)	
	Primary	9854	24.2%	2457 (24.9%)	7397 (75.1%)	
	No education	1100	2.7%	264 (24%)	836 (76%)	
Wealth quintile	Richer	3764	9.2%	1499 (39.8%)	2265 (60.2%)	.000
	Rich	5479	13.4%	1959 (35.8%)	3520 (64.2%)	
	Middle	7621	18.7%	2478 (32.5%)	5143 (67.5%)	
	Poor	11,006	27.0%	3317 (30.1%)	7689 (69.9%)	
	The poorest	12,881	31.6%	3375 (26.2%)	9506 (73.8%)	
Geographical domain	Jungle	10,954	26.9%	12,628 (31%)	28,123 (69%)	.000
	Highlands	13,609	33.4%	4814 (35.4%)	8795 (64.6%)	
	Rest of Coast	11,659	28.6%	3590 (30.8%)	8069 (69.2%)	
	Metropolitan Lima	4529	11.1%	1510 (33.3%)	3019 (66.7%)	
Area of residence	Urban	27,336	67.1%	8807 (32.2%)	18,529 (67.8%)	.000
	Rural	13,415	32.9%	3821 (28.5%)	9594 (71.5%)	
Health insurance coverage	Private insurance	478	1.2%	191 (40%)	287 (60%)	.000
	Armed or police forces	382	0.9%	140 (36.6%)	242 (63.4%)	
	ESSALUD	8311	20.4%	3130 (37.7%)	5181 (62.3%)	
	Public Health Insurance‐SIS	23,044	56.5%	6817 (29.6%)	16,227 (70.4%)	
	Uninsured	8536	20.9%	2350 (27.5%)	6186 (72.5%)	

*Note*: Demographic and Family Health Survey (ENDES 2019).

The use of oral health services was 31% and more prevalent (*p* = .000) in children aged 6–11 years (36.3%), belonging to the highest wealth quintile (39.8%), living in urban areas (32.2%), living in the geographical domain of the Highlands (35.4%), and enrolled in private health insurance (40%), as well as in children whose caregivers had higher education (37.8%) and received information on oral health care (40.2%). The only variable that did not show a statistically significant difference was the gender of the children (*p* = .077), and in all categories evaluated, the percentage of negative responses for the use of oral health services in the last 6 months was higher (Table 1).

Using the analysis for the calculation of prevalence ratios (PRs), the crude model reported a correlation between the use of oral health services and all variables except gender (*p* = .077). In relation to caregivers, primary education (*p* = .499) and age group 70 years (*p* = .062) did not correlate with their baseline variables. However, in the adjusted model, no correlation was found between the 50 and 69 age group (*p* = .085), being affiliated with the health insurance of the Armed Forces or Police versus no insurance (*p* = .188), and living in the geographical domain of the rest of the coast with respect to Metropolitan Lima (*p* = .967) (Table 2).

**Table 2:** Generalized linear model (RP) for the use of oral health services in children under 12 years old

Variable		Factors associated with the use of oral health services					
		Raw model			Adjusted model		
		PR	95% CI	*p* Value	PR	95% CI	*p* Value
Age	0–3 years						
	4–5 years	1.718	1.646–1.792	.000	1.677	1.610–1.748	.000
	6–11 years old	1.796	1.724–1.870	.000	1.846	1.775–1.921	.000
Gender	Female						
	Male	0.974	0.946–1.003	.077	0.973	0.947–1.000	.050
Caregiver received oral health information	No						
	Yes	2.555	2.456–2.658	.000	2.355	2.263–2.450	.000
Age of the person in charge	15–29 years old						
	30–49 years old	1.045	1.014–1.078	.004	0.944	0.916–0.973	.000
	50–69 years old	0.891	0.811–0.978	.015	0.932	0.852–1.021	.131
	70 years and older	0.675	0.447–1.020	.062	0.869	0.570–1.326	.516
Educational level of the caregiver	No education						
	Primary	1.039	0.930–1.160	.499	1.043	0.937–1,160	.440
	Secondary	1.264	1.135–1.407	.000	1.206	1.084–1.343	.001
	Higher education	1.575	1.414–1.754	.000	1.375	1.231–1.536	.000
Wealth quintile	The Poorest						
	Poor	1.150	1.104–1.198	.000	1.180	1.125–1.237	.000
	Middle	1.241	1.188–1.296	.000	1.225	1.156–1.298	.000
	Rich	1.365	1.303–1.429	.000	1.278	1.198–1.362	.000
	Richer	1.520	1.448–1.596	.000	1.323	1.232–1.421	.000
Geographical domain	Metropolitan Lima						
	Rest of Coast	0.924	0.879–0,970	.002	0.999	0.952–1.048	.967
	Highlands	1.061	1.012–1.112	.014	1.195	1.137–1.256	.000
	Jungle	0.743	0.705–0.783	.000	0.926	0.877–0.977	.005
Area of residence	Rural						
	Urban	1.131	1.096–1.168	.000	0.945	0.904–0.988	.013
Health insurance coverage	Uninsured						
	Public Health Insurance‐SIS	1.075	1.033–1.118	.000	1.112	1.069–1.158	.000
	ESSALUD	1.368	1.309–1.430	.000	1.166	1.117–1,218	.000
	Armed or Police Forces	1.331	1.162–1.526	.000	1.092	0.58–1.245	.188
	Private insurance	1.451	1.294–1.629	.000	1.124	1.001–1.262	.048

*Note*: Demographic and Family Health Survey (ENDES 2019).

The condition of having received information on oral health care (PRa = 2.355; 95% CI: 2.263–2.245) and affiliation with health insurance increased the likelihood of accessing these services. In the latter variable, belonging to a Private Health insurance scheme was more likely to receive care (PRa = 1.124; 95% CI: 1.001–1.262) than the Public Health Insurance (PRa = 1.112; 95% CI: 1.069–1.158). Urban children were less likely to attend oral health services (PRa= 0.945; 95% CI: 0.904–0.988) than those in rural areas, as were those in the geographical domain of the Jungle region (PRa = 0.926; 95% CI: 0.877–0.977) compared to Metropolitan Lima. On the other hand, the highest wealth quintile (RPa = 1.323; 95% CI: 1.232–1.421), the geographical domain of the Highlands (PRa = 1.195; 95% CI: 1.137–1.256), and the higher education level of the responsible person (PRa = 1.375; 95% CI: 1.231–1.536) also indicated to be factors that increase the probability of using dental services, when taking as reference the categories “The poorest,” “Metropolitan Lima,” and “No education,” respectively (Table 2).

Given the notorious correlation between children whose caregivers received information on oral health care and the use of dental services (PRa = 2.355; 95% CI: 2.263–2.245), an independent model was performed to evaluate this variable together with the factors related to caregivers. It was determined that those who received information about oral health care and belong to the highest wealth quintile (PR = 1.266; 95% CI: 1.173–1.366), as well as those who reached the highest educational level (PR = 1.332; 95% CI: 1.179–1.505), increased the probability of the use of oral health services of the children under their care, when taking as reference the categories “The poorest” and “No education,” respectively, while urban residents who received information presented lower probability of using them (PR = 0.945; 95% CI: 0.902–0.991) than rural residents (Table 3).

**Table 3:** Independent generalized linear (PR) model for oral health service use in children under 12 years of age whose caregivers received oral health care information

Variable	Category	Received information		
		PR	95% CI	*p* Value
Age of the caregiver	15–29 years old			
	30–49 years old	0.931	0.902–0.962	.000
	50–69 years old	0.956	0.866–1.057	.831
	70 years and older	0.568	0.292–1.104	.095
Educational level of the caregiver	No education			
	Primary	1.110	0.987–1.284	.083
	Secondary	1.192	1.059–1.341	.004
	Higher education	1.332	1.179–1.505	.000
Wealth quintile	The Poorest			
	Poor	1.116	1.061–1.174	.000
	Middle	1.167	1.097–1.241	.000
	Rich	1.214	1.133–1.300	.000
	Richer	1.266	1.173–1.366	.000
Geographical domain	Metropolitan Lima			
	Rest of Coast	1.025	0.974–1.079	.345
	Highlands	1.151	1.091–1.214	.000
	Jungle	0.957	0.903–1.014	.139
Area of residence	Rural			
	Urban	0.945	0.902–0.991	.018
Health insurance coverage	Uninsured			
	Public Health Insurance‐SIS	1.097	1.050–1.146	.000
	ESSALUD	1.131	1.079–1.185	.000
	Armed or police forces	1.066	0.927–1.227	.369
	Private insurance	1.905	0.968–1.239	.148

*Note*: Demographic and Family Health Survey (ENDES 2019).

## 4 DISCUSSION

The prevalence of children who visited a dentist in the last 6 months was less than one‐third of the sample included in the study; factors associated with the use of oral health services were having health insurance coverage, belonging to the highest quintiles of wealth, living in the Highlands and in Metropolitan Lima, living in a rural area, a higher level of education of the caregiver, and having received information on oral health care.

Estimates of the global burden of disease, reporting more than 530 million children diagnosed with dental caries (James et al., 2018), are consistent with the findings of the present study, in which only 31% of children under 12 years of age used oral health services in the 6 months before the survey date. However, according to 2019 National Report on Noncommunicable and Communicable Diseases, the percentage of children under 12 years of age seen in a dental service during 2017 and 2018 was 30.1% and 30.4%, respectively, while the difference compared to 2014 was 3.4 percentage points (Instituto Nacional de Estadística e Informática, 2019), which suggests a sustainable growth of the indicator and implies a positive effect of strategies aimed at improving children's oral health in the last 5 years, but which require optimization in their scope.

In this sense, it is noteworthy that in all the categories of the variables evaluated, the percentage of negative response for the use of oral health services was higher, which indicates a marked trend in the Peruvian population, also repeated in other regions (Gao et al., 2020; Nazir, 2018; Onyejaka et al., 2016; Portero de la Cruz & Cebrino, 2020). Most studies, seeking to explain this singularity, have shown that socio‐demographic factors have a different degree of influence on the attendance of the child population to oral health services depending on the place of residence (Alsuraim & Han, 2020; Aravena‐Rivas & Carbajal‐Rodríguez, 2021; Botello‐Harbaum et al., 2012; Fisher‐Owens et al., 2016; Hernández‐Vásquez et al., 2019), as in the meta‐analysis developed by Alsuraim & Han, which found an increase in the access of children under 12 years of age to dental services in countries with a lower level of development compared to more developed countries, due to the effect of globalization (Alsuraim & Han, 2020). This variability is consistent with the results related to the geographic domain of the Jungle, which recorded a lower probability of using oral health services than the inhabitants of Metropolitan Lima, as well as with the work of Aravena & Carbajal, who found that residents of the Jungle were less likely to seek dental care (Aravena‐Rivas & Carbajal‐Rodríguez, 2021). Similarly, Azañedo et al. identified fewer possibilities for its use in older adults living in the Jungle compared to Metropolitan Lima (Azañedo et al., 2019). This could be due to the greater proximity of the geographic domains of the Highlands and Metropolitan Lima, which facilitates transportation and the presence of dental services in the area, while in the Jungle, contact with the formal health system is conditioned by the limited connectivity links that exist.

Similarly, the finding of a lower probability of using dental services in urban versus rural areas has been reported in previous research (Aravena‐Rivas & Carbajal‐Rodríguez, 2021; Azañedo et al., 2019; Hernández‐Vásquez et al., 2019). It is possible to attribute this fact to the demographic dynamics, the increase of health facilities in this area after the implementation of the Universal Health Insurance, and the social programs of governmental financing, in spite of the higher index of poverty that includes the rural area (Hernández‐Vásquez et al., 2019; Ministerio de Salud, 2019b). However, given the influence of dietary habits on dental morbidity (Chaffee et al., 2017; Glick et al., 2021; Hobdell et al., 2003; Petersen et al., 2005; Zemaitiene et al., 2016), it is important to consider that, according to data on community nutritional status, in rural Peru, minors at school age consume more dairy products, fish, cereals, tubers, and legumes than in urban areas, as well as less meat, eggs, fruits, visible fats and high‐carbohydrate foods (López‐toledo et al., 2020). This nutritional pattern could reduce the incidence of dental caries among children in rural areas and explain a lower use of oral health services. When considering, for instance, that sugar consumption in urban areas reaches 43.41 g/day and in rural areas 18.58 g/day, while the obesity rate is 12% points higher in minors in urban areas (López‐toledo et al., 2020; Ministerio de Salud, 2019b). These data suggest that there are fewer risk factors for diseases of the oral cavity in children living in rural areas, although the national rate of malnutrition in children under 5 years of age in 2019 was 24.5% in rural areas and 7.6% in urban areas (Ministerio de Salud, 2019b), which implies a greater risk factor for painless conditions, such as enamel hypoplasia and gingivitis. Likewise, it should be considered that most studies show a higher prevalence of dental caries in minors in rural areas, but not in Peru, where the opposite occurs, although without a statistically significant difference (Gu et al., 2019; Ha et al., 2021; Ministerio de Salud, 2005). Therefore, it is possible that the conditions of poverty and remoteness, characteristics of rural Peruvian areas, are complemented by dietary habits and, therefore, reduce the perceived need for dental care by the rural population.

Likewise, although high levels of education appear as a positive constant, the most frequent reason for seeking oral health services is the presence of pain or discomfort, above preventive consultations, regardless of the educational level (Firmino, Martins, et al., 2018; Gao et al., 2020; Nazir, 2018; Quadri et al., 2018), which could explain why the oldest age group has presented the highest probability of care, as well as the mostly negative response to the use of oral health services even in the highest levels of wealth, with higher levels of education and informed about oral health care. On the other hand, the proportionate increase in prevalence by wealth quintiles and in patients with health coverage appears to be consistent with research that identifies high treatment costs, lack of health insurance, and lack of perceived need for dental treatment as the most common barriers that deter people from receiving dental care (Dho, 2018; Gao et al., 2020; Hernández‐Vásquez et al., 2019; Quadri et al., 2018). The data from these studies, together with the results of the present study, indicate that the implementation of the Universal Health Insurance Law has increased the use of dental services in the population and suggest that, in Peru, affiliation with health insurance and the nature of the insurance will be determining factors for its use.

For their part, Proaño & Bernabé determined that, during 2017, around 4% of Peruvian households incurred expenses above 40% of their ability to pay, within which dental care was found to be part of the second most onerous group of services (Proaño Falconi & Bernabé, 2018). Similarly, when evaluating the use of oral health services in the period 2004–2017, an increase was found that favored higher income groups (Hernández‐Vásquez et al., 2019). This trend is reflected in the correlation found for wealth quintiles, which found a higher likelihood for the “Richest” quintile to use oral health services than “The poorest” while progressively increasing for each quintile.

On a more specific range, there are studies reporting an association between knowledge about oral health care and the use of dental services in children. Quadri et al. found that patients who were informed about their oral health condition were 2 times more likely to keep their appointments, as were patients with a higher level of education, while patients who were advised by a dentist were 7 times more likely to keep their appointments (Quadri et al., 2018). Similarly, the relationship between adult caregivers receiving oral health care information and dental status in children was significant, both in the systematic review by Firmino, Ferreira et al. (2018) and in the studies by Adil et al. (2020) and Baskaradoss et al. (2019), although other reviews show contrary results (Firmino et al., 2017; Firmino, Martins, et al., 2018). In the present study, children whose caregivers received information on oral health care were more likely to use dental services, to the point of being the variable that showed the highest correlation. The results in Table 3 suggest that information sessions and campaigns have a better effect on the population with higher education and members of the highest wealth quintiles. It is possible that differences in educational standards within the country itself create inequities, considering the evidence that associates a lower level of education with difficulties in understanding therapeutic and preventive instructions, adhering to treatment regimens, and the tendency to develop inadequate health habits (Firmino et al., 2017). Similarly, the obstacles faced by lower‐income people in accessing the health care system are likely to contribute to barriers to dental care (Firmino, Martins, et al., 2018). Thus, in settings where there is a universal education system with uniform quality indicators and low values for indicators of social inequality, it is likely that having received information on oral health care is not significant; therefore, the heterogeneity of Peruvian population could explain these results.

Regarding oral health educational programs, the results are not conclusive (Firmino et al., 2017; Firmino, Martins, et al., 2018; George et al., 2019; Ghaffari et al., 2018; Goyal et al., 2019; Riggs et al., 2019; Xiao et al., 2019). The evidence of the impact of verbal presentations on oral health in the prevention of dental caries fluctuates in the range of low to very low certainty in pregnant females (George et al., 2019; Riggs et al., 2019; Xiao et al., 2019). However, in those interventions that included an interdisciplinary health team, home visits, motivational interviewing, and referrals for dental care as part of an institutional strategy, significant improvements were shown in clinical and behavioral oral health outcomes across the postnatal period (George et al., 2019). Similarly, research supporting the positive impact of oral health interventions in children indicate that motivational interviewing and community‐based programs are success factors (Ghaffari et al., 2018; Goyal et al., 2019; Xiao et al., 2019). Then, it is possible to notice that the methods to efficiently transmit oral health information to caregivers will be those that are incorporated in their periodic visits to health care facilities, motivational interviewing included in community‐based programs, and educational interventions in oral health integrated into mass media or common activities like school meetings.

Even so, the importance of the multifactorial approach to health prevention should not be diminished (Gao et al., 2020; George et al., 2019; Ghaffari et al., 2018; Goyal et al., 2019; Quadri et al., 2018). As shown by the results in Table 3, there was no statistically significant correlation between receiving information on oral health and those who only reached the primary level of education, while there was for higher levels. Evenly, having health coverage and belonging to higher wealth quintiles proved to be conditions for educational interventions to positively affect the use of oral health services. Thus, interventions that seek to improve oral health literacy may not have a significant impact if the SDOH remain unfavorable.

Considering the information presented and local research, which identifies the highest prevalence of childhood dental caries in the departments of Peru with the highest poverty index and the recording of its diagnosis as the second highest in outpatient consultations at the national level, with 42.5% of cases presented in children under 12 years of age (Gu et al., 2019; Instituto Nacional de Estadística e Informática, 2019; López‐toledo et al., 2020; Ministerio de Salud, 2019a, 2019b), the need to address inequity in the use of dental services through health strategies that are not implemented in isolation but in relation to policies aimed at changing the sociodemographic factors of the population becomes evident.

Among the limitations of the research, it is worth mentioning that the instrument used in the ENDES does not include an evaluation or report of the oral health status of the child, which does not allow us to evaluate the need for treatment or the impact of making use of health services. There is also the fact that causal relationships cannot be established, due to the cross‐sectional design of the study. However, the representative quality of the data obtained from the ENDES makes it possible to establish the correlation between the use of oral health services of children under 12 years of age, at the national level, with socio‐demographic factors, whose estimation has been standardized through a methodology validated by the INEI.

It is concluded that there is a low percentage of use of oral health services in Peruvian children under 12 years of age, despite the steady increase since 2014. Likewise, having health insurance, belonging to the middle‐income quintile and above, living in the Highlands, in Metropolitan Lima or in rural areas, a higher level of education of the person responsible, and having received information on oral health care predispose to the use of oral health services. It is worth noting that this last indicator was the one that presented the highest correlation and had the greatest impact on the population with the highest level of education and members of the highest wealth quintiles.

We recommend studies that address the need to evaluate the use of oral health services in each age group, including variables that consider the oral health status of the population, within their own demographics and social environment, to identify their specific limitations, given the proven variability in the relationships of socio‐demographic factors.

## AUTHOR CONTRIBUTIONS

José Diego Torres‐Mantilla and Edda E. Newball‐Noriega conceived the idea of this project. José Diego Torres‐Mantilla participated in data collection, data analysis, and wrote the initial draft of the paper. Edda E. Newball‐Noriega reviewed and edited the manuscript. Both authors approved the final version of the manuscript.

## CONFLICT OF INTEREST

The authors declare no conflict of interest.

## Data Availability

The data that support the findings of this study are available from the corresponding author, E. E. N‐N., upon reasonable request.
